# Post Mortem Artifacts by *Pheidole pallidula* (Hymenoptera: Formicidae) on a Human Corpse and an Overview of the Genus *Pheidole* in Forensic Entomology

**DOI:** 10.3390/insects17020180

**Published:** 2026-02-07

**Authors:** Marco Pezzi, Erica Di Biase, Federica Fumo, Domenico Bonelli, Federica Mendicino, Francesco Carlomagno, Enrico Schifani, Donato A. Grasso, Milvia Chicca, Vannio Vercillo, Teresa Bonacci

**Affiliations:** 1 Department of Chemical, Pharmaceutical and Agricultural Sciences, University of Ferrara, Via Luigi Borsari 46, 44121 Ferrara, Italy; 2 Department of Biology, Ecology and Earth Sciences, University of Calabria, Via Pietro Bucci, 87036 Rende, Italyfrancesco.carlomagno@unical.it (F.C.); teresa.bonacci@unical.it (T.B.); 3 Institute of Evolutionary Biology, CSIC—Pompeu Fabra University, Passeig Maritim de la Barceloneta 37–49, 08003 Barcelona, Spain; 4 Department of Chemistry, Life Sciences and Environmental Sustainability, University of Parma, Parco Area delle Scienze 11/A, 43124 Parma, Italy; donatoantonio.grasso@unipr.it; 5 Department of Life Sciences and Biotechnology, University of Ferrara, Via Luigi Borsari 46, 44121 Ferrara, Italy; milvia.chicca@unife.it; 6 Via Settimio Gargano, 87036 Rende, Italy; 7 Sistema Museale Universitario—SiMU, Sezione di Zoologia, University of Calabria, 87036 Rende, Italy

**Keywords:** Formicidae, forensics, injuries, Italy, *Pheidole*, skin post-mortem artifacts

## Abstract

Many ant species play a relevant role in the successional patterns of insects colonizing human and animal corpses. Although feeding behavior in ants depends on the trophic preferences of each taxon, these insects are often reported in several studies as predators of eggs, larvae, and adults of necrophagous insects. Among ants, some species are known to cause damage on human and animal dermis, known as post-mortem skin artifacts. The extensive activity of worker ants on corpses may hinder forensic pathological investigations aimed at determining the cause and location of death, as well as the assessment of entomologists in estimating the minimum post-mortem interval. We report for the first time a case of skin lesions on a human corpse found in a suburban area of the city of Cosenza (Calabria, Southern Italy), caused by the ant *Pheidole pallidula* (Hymenoptera: Formicidae). During the autopsy, numerous individuals (major and minor workers) were observed feeding on the corpse, but no other insects were found on it. We discuss the appearance of skin artifacts caused by *P. pallidula* and provide an overview of the genus *Pheidole* in forensic entomology.

## Simple Summary

We report for the first time a case of skin lesions due to active feeding of the ant *Pheidole pallidula* on a human corpse found in a suburban area of Cosenza (Region Calabria, Southern Italy). The autopsy revealed only major and minor workers of the species on the body, but no other insects. Ants are known to cause post-mortem skin damage on human and animal corpses and these damages may hinder pathologists’ forensic investigations into the cause, location and time of death. As this is the first case of skin artifacts caused on a human body by ants of the genus *Pheidole*, an overview of the relevance of the genus in forensic entomology is also provided.

## 1. Introduction

Some insects accelerate the decay process of corpses by feeding directly on the remains, while others, on the contrary, slow it down by preying on necrophagous insects [1]. Ants (Hymenoptera belonging to the family Formicidae) are widely distributed throughout the world [2] and are capable of both feeding on decaying remains and preying on other insects [3,4]. Thanks to their foraging strategies, efficient chemical recruitment and trail communication systems, many ant species can rapidly find, colonize and exploit new food resources from plants or animals (living or dead) [5,6,7,8]. These abilities enable ants to colonize animal carcasses and human remains, as confirmed by forensic evidence showing that ants may consume corpses, and interfere with dipteran colonizers used to estimate the minimum post-mortem interval by slowing the decomposition rate of corpses [3,9,10,11,12]. Feeding activity of ants may cause the formation of irregular superficial skin damages, which can be misinterpreted as ante-mortem injuries [3,10,13,14,15]. In Italy, the species of the family Formicidae reported to have caused post-mortem damages or artifacts on human bodies are *Tapinoma nigerrimum* (Nylander, 1856) in rural and suburban areas [10,13,15] and *Crematogaster scutellaris* (Olivier, 1791) in indoor areas [16]. Workers of *C. scutellaris* have also been observed preying on eggs and dipteran adults, causing damage to the skin of a pig carcass [9].

Here are reported for the first time skin lesions caused by the ant *Pheidole pallidula* (Nylander, 1849) on a human corpse found in a suburban area of the city of Cosenza (Calabria, Southern Italy). The appearance of skin artifacts caused by *P. pallidula* and their possible role in forensic crime scene alterations are also discussed, together with an overview of the involvement of the genus *Pheidole* in forensic entomology.

## 2. Case Report

**Figure 1:**
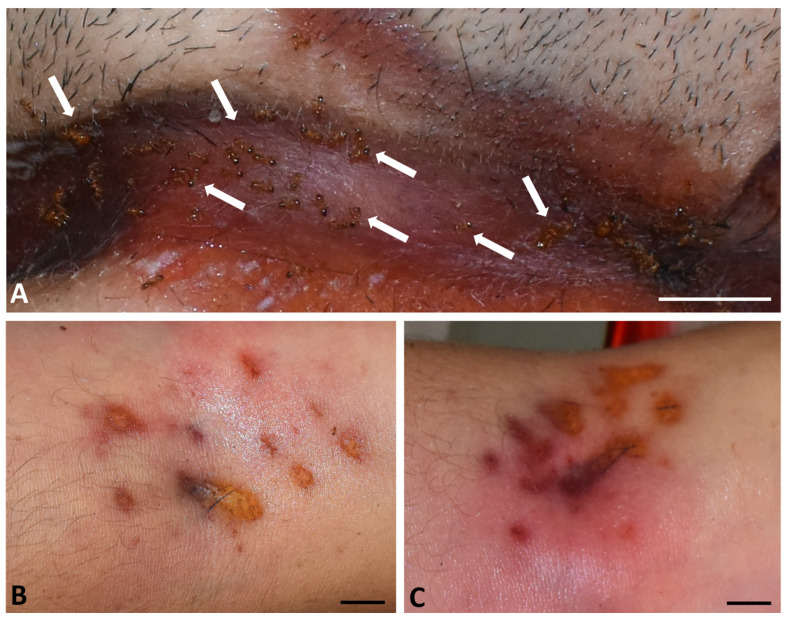
Autopsy of the deceased, external view. (**A**) Neck area: live ants (arrows) feeding in the lesions caused by hanging. Scale bar = 1.0 cm. (**B**) Skin lesions on the elbow crease of the left arm caused by ants. Scale bar = 0.5 cm. (**C**) Skin lesions on the elbow crease of the right arm caused by ants. Scale bar = 0.5 cm.

A 40-year-old man was found dead in a suburban area of Cosenza in August 2021. His body was discovered in the early morning in his home, hanging in a cellar that was locked from the inside. The man was wearing a T-shirt, shorts, and slippers. The autopsy, conducted three days after the body was discovered, determined the cause of death to be acute neurological and respiratory failure resulting from hanging. The presence of a hyoid bone fracture confirmed the hanging. Based on the findings and investigations conducted by the State Police, the post-mortem interval was estimated to be around 10–12 h, meaning the man died during the night. The case was officially declared a suicide. During the autopsy, live ant activity was observed post-mortem on the face, torso, back and arms, as well as on eyes and eyelids. The ants were observed feeding and moving on the skin surface (Figure 1). Macroscopic examination revealed irregular yellow-orange and brown lesions (0.5–5 cm long), scattered across the skin of the neck at the level of the nylon string used for hanging. No fluid or blood loss was observed on the damaged skin. Ants were observed feeding near the lesions and along the edges of the neck wounds (Figure 1A). Additional reddish-brown and roundish skin lesions (0.2 to 1.1 cm long) were scattered across the skin surface of the elbow crease of both arms (Figure 1B,C).

A total of 13 live ants from all those found alive on the body were collected during the autopsy with entomological tweezers and preserved in 70% alcohol for species identification. During the ant sampling, no larvae or dipteran eggs were found on the body, probably because the death occurred at night and in a cellar with limited access to flies.

Individuals were observed and photographed using a stereomicroscope (Meiji Techno RZ, Meiji Techno America, Santa Clara, CA, USA), equipped with an image acquisition system (Delta Pix camera, Smoerum, Denmark), and then identified using taxonomic articles and books [17,18,19,20]. All collected ants were preserved in 70% alcohol at the Department of Biology, Ecology and Earth Sciences of the University of Calabria (Rende, Italy).

Histological analyses were performed on skin lesions caused by live ants on the right arm of the deceased (Figure 1C), according to a previously published protocol [15]. Sections were observed under a light microscope (Leica DM4, Leica Microsystems, Wetzlar, Germany) and acquired with a digital camera (Leica Microsystems, Wetzlar, Germany).

## 3. Bibliographic Methods

The bibliographic search on ants in forensic entomology was initially based on a previous study [15] and on publications indexed on PubMed, and was subsequently extended through web engines using the keywords “ants, artifacts, Formicidae, forensic entomology, animal and human corpses, *Pheidole*, *Pheidole pallidula*”. In addition to case reports, this search led to another review [21], which included a list of carrion-associated ant species and their references. The search about the role of *Pheidole pallidula* and the genus *Pheidole* in forensic entomology was conducted through November 2025.

## 4. Results and Discussion

### 4.1. Species Identification

Among European ants, identifying the genus *Pheidole* is relatively simple, based on the morphology of the mesosoma, node, and head. Females have antennae composed of 12 segments, with the last three antennomeres forming a club. Workers are dimorphic, with majors (also known as soldiers) possessing a massive head to produce an increased mandibular force [18,19,22].

In Italy, two species of the genus *Pheidole* have been reported outdoors. The first species, present in all Italian territory, is *P. pallidula*, belonging to the *P. pallidula* complex, including *P. pallidula*, *P. balcanica* Seifert, 2016, *P. koshewnikovi* Ruzsky, 1905 and *P. cicatricosa* Stitz, 1917 [17]. The second species is the non-native *Pheidole indica* (Mayr 1879), known mainly from urban areas of Sicily and the nearby island of Pantelleria [23,24,25]. Two other non-native species have been reported, *Pheidole megacephala* (Fabricius, 1793), found in 2001 in greenhouses of plants imported from Asia in Parabiago (Milan) and in 2006 inside a storage hangar in Cargo City (Milan Malpensa Airport, Ferno, Varese, Italy), and *Pheidole nodus* Smith, 1874, found in 2001 in greenhouses of plants imported from Asia in Parabiago (Milan). Neither species has been confirmed as established [25,26,27].

**Figure 2:**
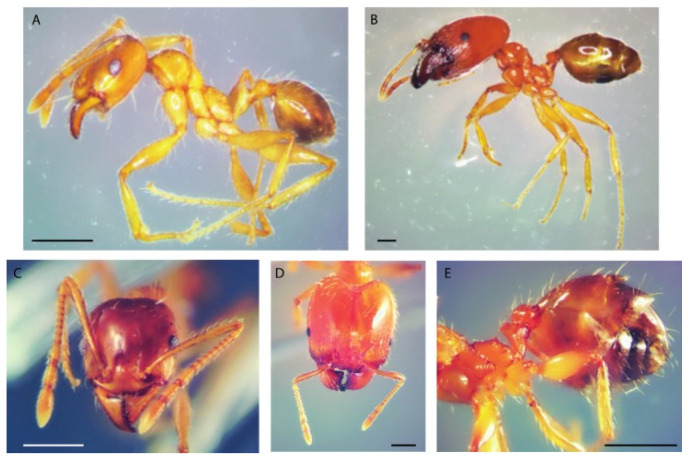
Individuals of *Pheidole pallidula* collected on the deceased. (**A**) Minor worker, lateral view. (**B**) Major worker, lateral view. (**C**) Head of a minor worker, frontal view. (**D**) Head of a major worker, frontal view. (**E**) Major worker. From left to right: mesosoma, petiole, postpetiole and gaster. Scale bars = 0.5 mm.

Workers of the *P. pallidula* complex are easily distinguishable from *P. indica* by the lack of a deep promesonotal groove and by the smoother head sculpture of soldiers, and from *P. megacephala* by the different ventral profile of the postpetiole. Morphological analysis of the collected ants confirmed that all specimens belonged to the *Pheidole pallidula* complex (Figure 2). Since *P. pallidula* is the only species of the complex recorded in Italy, the collected specimens are attributed to this species.

*Pheidole pallidula* is an opportunistic, often dominant, omnivorous species, known to feed on invertebrates, seeds, and honeydew; it generally nests in ground or under stones in colonies of tens of thousands of individuals [18,19].

### 4.2. Case Interpretation and Literature Overview

Ants have been documented as playing a role in sarcosaprophagous insect communities and have been found associated with animal and human corpses in various habitats across several geographical areas, both outdoors and indoors.

The role of ants in sarcosaprophagous insect communities has been documented, and they have been found associated with animal and human corpses in various habitats across different geographic areas, both indoors and outdoors. A recent extensive study reviews in detail the role of ants in vertebrate carrion decomposition [21]. Like other insects, ants can influence the decay process of dead bodies by feeding on remains, preying on carrion arthropods, or using corpses as nesting sites or refuges [21,28,29]. Ant feeding activities on corpses can produce post-mortem artifacts on the skin of humans and animals [3,10,11,13,15,21,30].

These post-mortem artifacts typically appear as serpiginous lesions on the skin surface of many anatomical areas [3,10,30]. Post-mortem damage inflicted by unidentified species of the genera *Atta* and *Cephalotes* have been reported in Brazil [31], with the former genus found in rural and urban areas and the latter genus in forest areas. Other unidentified Formicidae taxa have been reported to inflict post-mortem damage on human skin [15].

In Italy, the ant species reported to have caused post-mortem damages or artifacts on human bodies are *T. nigerrimum* in rural and suburban areas [10,13,15], now considered a species complex [25] and *C. scutellaris* indoors [16].

Among the Formicidae taxa feeding on corpses, the cosmopolitan genus *Pheidole* Westwood, 1839, belonging to the subfamily Myrmicinae, has historically been reported as occasionally present on carcasses, with an unclear ecological role [32]. This genus currently includes over 1160 described and more than 1500 estimated species, thus representing one of the most species-rich ant genera, together with the genus *Camponotus* Mayr, 1861 [18,33,34].

Within the genus *Pheidole*, 20 species and 1 subspecies of the genus have been associated with vertebrate carcasses, ranging from small animals, such as chicks and mice, to large ones, such as pigs, deer and American alligators (Table 1). Other reports mention the genus *Pheidole* in association with vertebrate carcasses, but without species identification (Table 2). Individuals belonging to the genus, with or without species identification, have also been captured in necrophagous insect traps loaded with pig and cow liver, chicken meat and sardines (Table 1 and Table 2). In Italy, the only report of possible necrophagous activity of the genus *Pheidole* concerned pig carcasses periodically exhumed on the succession of arthropod fauna in experimental studies on decomposition stages in Calabria [35].

The ecological role of species of the genus *Pheidole* apparently ranges from necrophagous to predatory and omnivorous. In Brazil, *Pheidole radoszkowskii* Mayr, 1884, has been reported to cause skin lesions on rat carrions [36], and an unidentified species of the genus *Pheidole* was reported to cause small lesions on nose and external ear of a rabbit [37]. In Malaysia, *P. megacephala* was found associated with exposed rat and macaque carcasses and was thought to be responsible for slowing the decomposition rate of the examined animals [12]. Environments where the genus *Pheidole* was found associated with carcasses and entomological traps were forests, urban, rural and suburban areas (Table 1 and Table 2). To date, the genus *Pheidole* has never been reported actively feeding on human corpses anywhere in the world. The case reported here in Southern Italy is therefore the first involving necrophagous activity on a human corpse of the genus *Pheidole* and specifically of *P. pallidula*. This species had previously been mentioned as associated with animal carcasses and traps in open environments (Table 1), so this is also the first case reporting active feeding of *P. pallidula* on a human corpse indoors.

**Table 1:** Cases involving species and subspecies of the genus *Pheidole* detected on animal corpses, on animal meat in entomological traps, and on a human corpse. NR, not reported.

	Species/Subspecies	Corpses/Traps	Reported Behavior	Environment	Country	References
1	*Pheidole pallidula*	Pig	Necrophilous	Rural	Spain	[38]
		Chicken carcass (trap)	NR	Rural	Spain	[39,40]
		Pig	NR	Forest, maquis, bush	Spain	[41]
		Human	Necrophagous	Suburban	Italy	Present report
2	*Pheidole alienata*	Pig	Omnivorous	Forest	Brazil	[14]
3	*Pheidole bicarinata vinelandica*	Pig	Predator	Forest	United States (South Carolina)	[42]
4	*Pheidole crassinoda*	Impala	Sarcophagous, saprophagous, predator, opportunistic	Forest (woodland savannah)	South Africa	[43]
5	*Pheidole dentata*	Swine, Deer, American alligator	NR	Forest	United States (Louisiana)	[44]
		Dog	Omnivorous	Forest, pasture	United States (Tennessee)	[45]
		Chick (trap)	NR	Forest	United States (Florida)	[46]
6	*Pheidole fallax*	Pig	Omnivorous	Forest	Brazil	[14]
7	*Pheidole fervida*	Pig	Predator	Suburban	South Korea	[47]
		Rabbit	NR	Suburban	South Korea	[48]
8	*Pheidole flavens*	Pig	Predator	Forest	Brazil	[14]
9	*Pheidole funkikoensis*	Pig liver (trap)	Necrophilous	Forest	Taiwan	[49]
10	*Pheidole hyatti*	Pig	NR	Urban	Mexico	[50]
11	*Pheidole indica*	Pig liver (trap)	Necrophilous	Urban	Taiwan	[49]
		Pig	NR	Suburban	India	[51]
12	*Pheidole liengmei*	Impala	Sarcophagous, saprophagous, predator, opportunistic	Forest (woodland savannah)	South Africa	[43]
13	*Pheidole longipes*	Macaque	NR	Forest	Malaysia	[52]
14	*Pheidole megacephala*	Pig liver (trap)	Necrophilous	Forest and urban	Taiwan	[49]
		Rat, macaque, rabbit	NR	Urban forest	Malaysia	[53]
		Rat, macaque	Necrophagous, predator	Urban forest	Malaysia	[12]
		Rabbit	Necrophagous and predator	Urban	Egypt	[54]
		Rabbit	Predator	Rural	Kingdom of Saudi Arabia	[55]
		Rabbit	NR	Rural	Kingdom of Saudi Arabia	[56]
		Cat	Predator	Suburban	United States (Hawaii)	[57]
		Cow liver, grazing chicken, chicken, sardine	Necrophagous and predator	Urban	Iraq	[58]
15	*Pheidole midas*	Pig	Predator	Forest	Brazil	[14]
16	*Pheidole moerens*	Chick (trap)	NR	Forest	United States (Florida)	[46]
17	*Pheidole nodus* (syn. *Pheidole formosensis*; syn. *Pheidole noda*)	Mouse (trap)	Necrophilous	Forest, meadow	Taiwan	[59]
		Pig liver (trap)	Necrophilous	Forest and urban	Taiwan	[49]
18	*Pheidole parva* (syn. *Pheidole sauteri*)	Mouse (trap)	Necrophilous	Not reported	Taiwan	[59]
19	*Pheidole pieli*	Mouse (trap)	Necrophilous	Not reported	Taiwan	[59]
20	*Pheidole radoszkowskii*	Pig	Omnivorous	Forest	Brazil	[14]
		Rat *	Necrophagous and predator	Urban	Brazil	[36]
		Pig	NR	Urban	Brazil	[60]
21	*Pheidole ryukyuensis*	Pig liver (trap)	Necrophilous	Urban	Taiwan	[49]
22	*Pheidole sitarches*	American alligator	NR	Forest	United States (Louisiana)	[44]
23	*Pheidole synarmata*	Pig	NR	Urban	Brazil	[60]
		Pig	NR	Urban forest	Brazil	[61]

* Skin alterations reported, without detailed description.

**Table 2:** Cases involving the genus *Pheidole* (without species identification) detected on animal corpses and in entomological traps. NR, not reported.

Animal Corpses/Traps	Reported Behavior	Environment	Country	References
Dog	Omnivorous	Forest, pasture	United States (Tennessee)	[45]
Fish (trap) Chicken gizzard (trap)	Necrophagous and predator	Forest	Brazil	[62]
Pig	NR	Forest	Brazil	[63]
Pig	Omnivorous (necrophagous and predator)	Rural	Brazil	[64]
Pig	Omnivorous	Urban	Colombia	[65]
Pig	Necrophagous and predator	Urban	Nigeria	[66]
Pig	Predator	Rural	Ghana	[67]
Pig	NR	Rural	Italy	[35]
Pig	Necrophagous and predator	Rural	Brazil	[68]
Rabbit	NR	Urban	Zimbabwe	[69]
Rabbit **	Necrophagous and predator	Rural	Brazil	[37]
Rat	NR	Rural	Brazil	[70]
Rat	NR	Urban	Cameroon	[71]
Rat, macaque, rabbit	Predator	Forest	Malaysia	[53]
Vertebrates	Unclear	NR	Australia	[32]

** Small lesions on the nose and the external ear.

Injuries caused by ants and other arthropods [15] could be mistaken for those resulting from chemical burns, cigarette scars, nail abrasions or intravenous drug use [3], making it crucial to distinguish them for accurate crime scene reconstruction. In the reported case, the first interpretation of the injuries on the arms by investigators was that they were repeated intravenous injections.

**Figure 3:**
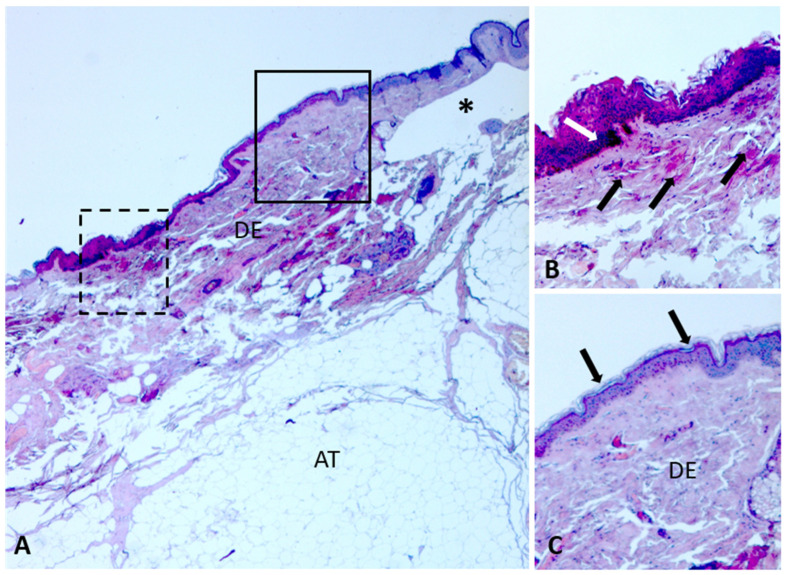
Histological analyses on post-mortem lesions caused by *Pheidole pallidula* on skin of the right arm of the deceased. Sections stained with Masson’s trichrome. (**A**) Section showing parts with lesions caused by feeding activity of *P. pallidula*. The asterisk indicates an anomalous dermal blister. (**B**) Detail of the dashed area in (**A**), showing skin surface with hyperkeratosis and areas with hypereosinophilia (white arrow) and vascular activation (black arrows). (**C**) Detail of the outlined area in (**A**), showing intact skin and regular inter-papillary ridges (arrows). Abbreviations: AT, adipose tissue; DE, dermis.

Although the morphology of the lesions cannot yet be considered species-specific because the data are still indicative, some differences can be appreciated by comparing the images of lesions caused by *P. pallidula* with those produced by *T. nigerrimum* complex [13,15]. In *P. pallidula*, the lesions appear roundish-elliptical in shape, with smooth and non-serpiginous margins, while those caused by *T. nigerrimum* complex appear irregular and serpiginous. Preliminary histological analyses revealed that the lesions inflicted by *P. pallidula* and *T. nigerrimum* complex [15] are both characterized by anomalous blister formation (Figure 3A). The blisters caused by *P. pallidula* appear located in the upper dermis and those by *T. nigerrimum* complex in the lower dermis. Moreover, the lesions caused by *P. pallidula* apparently include areas of hyperkeratosis, hypereosinophilia, and vascular activation (Figure 3B), compared with adjacent intact skin (Figure 3C), while these areas are apparently absent in lesions induced by *T. nigerrimum* complex [15].

Although the case involving *P. pallidula* is a single one, the preliminary results suggest a different way of skin consumption in the two species. Further studies will be needed to evaluate the chemical composition of *P. pallidula* glands and the potential role of enzymes in pre-oral digestion.

In addition to their importance for studies on the biology of the species, investigations of post-mortem skin damage caused by *P. pallidula* can help avoid misinterpretations of these lesions during forensic investigations. Although ants have been clearly recognized as part of the sarcosaprophagous community, their role in producing skin artifacts is still poorly understood and often underestimated in forensic practice.

Our data suggest the need for in-depth investigations to identify the origin of any injuries that may be caused by macro- and microfauna after death. Studies that integrate entomological expertise with forensic pathology will be essential to establish diagnostic criteria for differentiating ant-induced lesions and improve the accuracy of estimating the post-mortem interval and reconstructing the death scene. Research involving experiments with animal models in controlled indoor and outdoor environments will also be useful to obtain more details on differences in feeding activity and the morphology of lesions caused by ants on corpses, in order to develop more standardized diagnostic criteria.

## Data Availability

The original contributions presented in this study are included in the article. Further inquiries can be directed to the corresponding author.

## References

[1] Smith K.G.V. (1986). A Manual of Forensic Entomology.

[2] Gibb H., Dunn R.R., Sanders N.J., Grossman B.F., Photakis M., Abril S., Agosti D., Andersen A.N., Angulo E., Armbrecht I. (2017). A global database of ant species abundances. Ecology.

[3] Campobasso C.P., Marchetti D., Introna F., Colonna M.F. (2009). Postmortem artifacts made by ants and the effect of ant activity on decompositional rates. Am. J. Forensic Pathol..

[4] Byrd J.H., Castner J.L. (2010). Forensic Entomology: The Utility of Arthropods in Legal Investigations.

[5] Grasso D.A., Mori A., Le Moli F. (1998). Chemical communication during foraging in the harvesting ant *Messor capitatus* (Hymenoptera, Formicidae). Insectes Sociaux.

[6] Grasso D.A., Mori A., Le Moli F. (1999). Recruitment and trail communication in two species of *Messor* ants (Hymenoptera, Formicidae). Ital. J. Zool..

[7] Visicchio R., Mori A., Grasso D.A., Castracani C., Le Moli F. (2001). Glandular sources of recruitment, trail, and propaganda semiochemicals in the slave-making ant *Polyergus rufescens*. Ethol. Ecol. Evol..

[8] Scarano F., Giannetti D., Trenti F., Giacomazzi F., Vigna J., Guella G., Grasso D.A., Haase A. (2024). Trail pheromone identification in the ant *Crematogaster scutellaris*. Sci. Rep..

[9] Bonacci T., Zetto Brandmayr T., Brandmayr P., Vercillo V., Porcelli F. (2011). Successional patterns of the insect fauna on a pig carcass in southern Italy and the role of *Crematogaster scutellaris* (Hymenoptera, Formicidae) as a carrion invader. Entomol. Sci..

[10] Bonacci T., Benecke M., Scapoli C., Vercillo V., Pezzi M. (2019). Severe post mortem damages by ants on a human corpse. Rom. J. Leg. Med..

[11] Ramón-Cabrera G., Donoso D.A. (2015). The role of ants (Hymenoptera: Formicidae) in forensic entomology. Rev. Ecuat. Med. Y Cienc. Biológicas.

[12] Ahmad A., Omar B. (2023). A report of *Pheidole megacephala* (Fabricius 1793) (Hymenoptera: Formicidae) colonisation on small and medium-sized mammal carcasses: Its implications in forensic entomology. Int. J. Trop. Insect Sci..

[13] Bonacci T., Vercillo V. (2015). Outdoor post-mortem bite injuries by *Tapinoma nigerrimum* (Hymenoptera, Formicidae) on a human corpse: Case report. J. Forensic Leg. Med..

[14] Paula M.C., Morishita G.M., Cavarson C.H., Gonçalves C.R., Tavares P.R.A., Mendonça A., Súarez Y.R., Antonialli-Junior W.F. (2016). Action of ants on vertebrate carcasses and blow flies (Calliphoridae). J. Med. Entomol..

[15] Pezzi M., Benecke M., Vercillo V., Marino F., Marino S., Scapoli C., Marchetti M.G., Leis M., Bonacci T. (2020). Histochemical and immunohistochemical investigations on postmortem injuries caused by ants and literature review. Rom. J. Leg. Med..

[16] Bugelli V., Forni D., Bassi L.A., Di Paolo M., Marra D., Lenzi S., Toni C., Giusiani M., Domenici R., Gherardi M. (2015). Forensic entomology and the estimation of the minimum time since death in indoor cases. J. Forensic Sci..

[17] Seifert B. (2016). Inconvenient hyperdiversity–the traditional concept of “*Pheidole pallidula*” includes four cryptic species (Hymenoptera: Formicidae). Soil Org..

[18] Seifert B. (2018). The Ants of Central and North Europe.

[19] Lebas C., Galkowski C., Blatrix R., Wegnez P. (2019). Ants of Britain and Europe: A Photographic Guide.

[20] Lenoir A., Perdereau E., Dupont S., Libbrecht R., Picquet P., Galkowski C., Mercier J.-L. (2025). The tropical ant *Pheidole megacephala* (Fabricius, 1793) in France and discrimination with the local *Pheidole pallidula* (Nylander, 1849). Naturae.

[21] Eubanks M.D., Lin C., Tarone A.M. (2019). The role of ants in vertebrate carrion decomposition. Food Webs.

[22] Wilson E.O. (2003). Pheidole in the New World: A Dominant, Hyperdiverse Ant Genus.

[23] Mei M. (1995). Hymenoptera Formicidae (con diagnosi di due nuove specie). Nat. Sicil..

[24] Schifani E., Alicata A. (2018). Exploring the myrmecofauna of Sicily: Thirty-two new ant species recorded, including six new to Italy and many new aliens (Hymenoptera, Formicidae). Pol. J. Entomol..

[25] Schifani E. (2022). The new checklist of the Italian Fauna: Formicidae. Biogeographia.

[26] Limonta L., Colombo M. (2003). Record of *Pheidole megacephala* (F.), *Pheidole nodus* Smith and *Tetramorium bicarinatum* Nylander (Hymenoptera Formicidae), tropical species, in nursery imported plants. Boll. Zool. Agrar. Bachic..

[27] Jucker C., Rigato F., Regalin R. (2008). Exotic ant records from Italy (Hymenoptera, Formicidae). Boll. Zool. Agrar. Bachic..

[28] Payne J.A. (1965). A summer carrion study of the baby pig *Sus scrofa* Linnaeus. Ecology.

[29] Forbes S.L., Carter D.O. (2015). Processes and Mechanisms of Death and Decomposition of Vertebrate Carrion.

[30] Erkol Z., Hösükler E., Doga K.H. (2018). Postmortem animal attacks on human corpses. Post Mortem Examination and Autopsy—Current Issues from Death to Laboratory Analysis.

[31] de Souza A.A.D.F., de Rosa C.T.A., Arantes L.C., Pujol-Luz J.R. (2020). Artifacts caused by leaf-cutting ants of the genus *Atta* (Hymenoptera: Formicidae): Postmortem bite injuries and the tearing of clothes. J. Forensic Sci..

[32] Fuller M.E. (1934). The Insect Inhabitants of Carrion: A Study in Animal Ecology.

[33] Bolton B. (2025). An Online Catalog of the Ants of the World. https://antcat.org.

[34] Oberski J.T., Griebenow Z.H., Adams R.M.M., Andersen A., Andrade-Silva J., Barden P., Borowiec M., Brady S., Casadei-Ferreira A., Csősz S. (2025). Ant systematics: Past, present, and future. Insect Syst. Divers..

[35] Bonacci T., Mendicino F., Bonelli D., Carlomagno F., Curia G., Scapoli C., Pezzi M. (2021). Investigations on arthropods associated with decay stages of buried animals in Italy. Insects.

[36] de Sales T.A., Daemon E., Lopes J.F.S. (2015). Necrophagous or predators? The role of *Pheidole radoszkowskii* Mayr (Hymenoptera: Formicidae) on *Rattus norvegicus* (Berkenhout) carcasses (Rodentia: Muridae). J. Nat. Hist..

[37] de Souza A.S.B., Kirst F.D., Krüger R.F. (2008). Insects of forensic importance from Rio Grande do Sul state in southern Brazil. Rev. Bras. Entomol..

[38] Castillo Miralbés M. (2002). Estudio de la entomofauna asociada a cadáveres en el alto Aragón (España). Monogr. Soc. Entomológica Aragonesa.

[39] Martínez M.D., Arnaldos M.I., Romera E., García M.D. (2002). Los Formicidae (Hymenoptera) de una comunidad sarcosaprófaga en un ecosistema mediterráneo. Ann. Biol..

[40] Arnaldos M.I., Romera E., Presa J.J., Luna A., García M.D. (2004). Studies on seasonal arthropod succession on carrion in the southeastern Iberian Peninsula. Int. J. Leg. Med..

[41] Pérez-Marcos M., López-Gallego E., Arnaldos M.I., Martínez-Ibáñez D., García M.D. (2020). Formicidae (Hymenoptera) community in corpses at different altitudes in a semiarid wild environment in the southeast of the Iberian Peninsula. Entomol. Sci..

[42] Payne J.A., Mason W.R.M. (1971). Hymenoptera associated with pig carrion. Proc. Entomol. Soc. Wash.

[43] Braack L.E.O. (1986). Arthropods associated with carcasses in the northern Kruger National Park. S. Afr. J. Wildl. Res..

[44] Watson E.J., Carlton C.E. (2005). Insect succession and decomposition of wildlife carcasses during fall and winter in Louisiana. J. Med. Entomol..

[45] Reed H.B. (1958). A study of dog carcass communities in Tennessee, with special reference to the insects. Am. Midl. Nat..

[46] Scott M.P., Traniello J.F.A., Fetherston I.A. (1987). Competition for prey between ants and burying beetles (*Nicrophorus* spp): Differences between Northern and Southern temperate sites. Psyche.

[47] Park S.-H., Moon T.-Y. (2020). Positive and negative impact to decomposition of pig cadavers by ants (Hymenoptera: Formicidae) at the Islet Yeongdo in Busan, South Korea. Entomol. Res..

[48] Park S.-H., Moon T.-Y. (2020). Forensic implication of cadaveric behaviors of the Japanese pavement ant (*Tetramorium tsushimae* Emery) attending to rabbit cadavers. Entomol. Res..

[49] Leong C.M., Shelomi M., Lin C.C., Shiao S.-F. (2019). Necrophilous ants (Hymenoptera: Formicidae) in diverse habitats in Taiwan. Sociobiology.

[50] Valdes-Perezgasga M.T., Sanchez-Ramos F.J., Garcia-Martinez O., Anderson G.S. (2010). Arthropods of forensic importance on pig carrion in the Coahuilan semidesert, Mexico. J. Forensic Sci..

[51] Bala M., Kaur P. (2015). Insect faunal succession on buried piece of pork in the State of Punjab (India): A preliminary study. J. Forensic Res..

[52] Chen C.D., Nazni W.A., Lee H.L., Hashim R., Abdullah N.A., Ramli R., Lau K.W., Heo C.C., Goh T.G., Izzul A.A. (2014). A preliminary report on ants (Hymenoptera: Formicidae) recovered from forensic entomological studies conducted in different ecological habitats in Malaysia. Trop. Biomed..

[53] Azwandi A., Nina Keterina H., Owen L.C., Nurizzati M.D., Omar B. (2013). Adult carrion arthropod community in a tropical rainforest of Malaysia: Analysis on three common forensic entomology animal models. Trop. Biomed..

[54] Tantawi T.I., El-Kady E.M., Greenberg B., El-Ghaffar H.A. (1996). Arthropod succession on exposed rabbit carrion in Alexandria, Egypt. J. Med. Entomol..

[55] Shaalan E.A., El-Moaty Z.A., Abdelsalam S., Anderson G.S. (2017). A preliminary study of insect succession in Al-Ahsaa Oasis, in the eastern region of the Kingdom of Saudi Arabia. J. Forensic Sci..

[56] Al-Dakhil A.A., Alharbi S.A. (2020). A preliminary investigation of the entomofauna composition of forensically important necrophagous insects in Al-Madinah Al-Munawwarah region, Kingdom of Saudi Arabia. J. Taibah Univ. Sci..

[57] Early M., Goff M.L. (1986). Arthropod succession patterns in exposed carrion on the island of O’ahu, Hawaiian Islands, USA. J. Med. Entomol..

[58] Al-Ameri D.T., Hamza A.K., Alhasan A.S. (2020). Relationship between ants *Pheidole megacephala* (Hymenoptera: Formicidae) and some dead animals tissue. Plant Arch..

[59] Hwang W. (2011). Environmental variables affecting community composition and carcass utilization of necrophagous ant (Hymenoptera: Formicidae) in Southern and Northern Taiwan. BioFormosa.

[60] Andrade-Silva J., Pereira E.K.C., Silva O., Santos C.L.C., Delabie J.H.C., Rebêlo J.M.M. (2015). Ants (Hymenoptera: Formicidae) associated with pig carcasses in an urban area. Sociobiology.

[61] Pereira E.K.C., Andrade-Silva J., Silva O., Santos C.L.C., Moraes L.S., Bandeira M.d.C.A., Silva C.R.R., Rebêlo J.M.M. (2017). *Solenopsis saevissima* (Smith) (Hymenoptera: Formicidae) activity delays vertebrate carcass decomposition. Sociobiology.

[62] Moretti T.C., Solis D.R., Godoy W.A.C. (2013). Ants (Hymenoptera: Formicidae) collected with carrion-baited traps in Southeast Brazil. Open Forensic Sci. J..

[63] Cruz T.M., Vasconcelos S.D. (2006). Entomofauna de solo associada à decomposição de carcaça de suíno em um fragmento de Mata Atlântica de Pernambuco, Brasil. Biociências.

[64] Viana G.S., de Paula M.C., de Moura Eulalio A.D., dos Santos P.G., Lima-Junior S.E., Antonialli-Junior W.F. (2022). Formicidae fauna in pig carcasses contaminated by insecticide: Implications for forensic entomology. Rev. Bras. Entomol..

[65] Wolff M., Uribe A., Ortiz A., Duque P. (2001). A preliminary study of forensic entomology in Medellín, Colombia. Forensic Sci. Int..

[66] Ekanem M.S., Dike M.C. (2010). Arthropod succession on pig carcasses in Southeastern Nigeria. Pap. Avulsos Zool.

[67] Kyerematen R.A.K., Boateng B.A., Haruna M., Eziah V.Y. (2013). Decomposition and insect succession pattern of exposed domestic pig (*Sus scrofa* L.) carrion. ARPN J. Agric. Biol. Sci..

[68] Gomes L., Gomes G., Desuó I.C. (2009). A preliminary study of insect fauna on pig carcasses located in sugarcane in winter in south-eastern Brazil. Med. Vet. Entomol..

[69] Mabika N., Masendu R., Mawera G. (2014). An initial study of insect succession on decomposing rabbit carrions in Harare, Zimbabwe. Asian Pac. J. Trop. Biomed..

[70] Monteiro-Filho E.L.A., Penereiro J.L. (1987). Estudo da decomposição e sucessão sobre uma carcaça animal numa área do estado de São Paulo, Brasil. Rev. Bras. Biol..

[71] Feugang Youmessi F.D., Bilong Bilong C.F., Cherix D., Djiéto-Lordon C. (2012). Biodiversity study of arthropods collected on rat carrion in Yaounde, Cameroon: First study on forensic entomology in Central Africa. Int. J. Biosci..

